# Microstructure and Properties of TiCp/Fe Hierarchical Composites Prepared by a New Pressure Infiltration Method

**DOI:** 10.3390/ma17061325

**Published:** 2024-03-13

**Authors:** Shengnian Zhao, Dehong Lu, Fengbin Wang, Jiaxing Zhong, Yehua Jiang

**Affiliations:** 1 Faculty of Materials Science and Engineering, Kunming University of Science and Technology, Kunming 650093, China; 18298343639@163.com (S.Z.); 15797634416@163.com (F.W.); 13330496952@163.com (J.Z.); jiangyehua@kmust.edu.cn (Y.J.); 2 National and Local Joint Engineering Research Center for Advanced Solidification Forming and Equipment Technology of Metals, Kunming 650093, China

**Keywords:** pressure infiltration, hierarchical architectured composite, interface, mechanical properties, fracture behavior

## Abstract

TiCp/steel composites are conventionally produced via powder metallurgy. In this paper, a liquid pressure infiltration method was developed to prepare a kind of spherical hierarchical architectured composite, in which spherical TiCp-rich hard phase regions were uniformly dispersed in TiCp-free soft phase region. The microstructure and mechanical properties of the architectured composites were carefully studied and compared with the common composite, as well as the effect of TiCp fraction on the properties. The results show that architecturual design can effectively improve both the toughness and strength of the composites. With TiCp content increasing from 30% to 50%, both the bending strength and the impact toughness of the architectured composites first increase, then decrease, and reach the highest at 40% TiCp. The highest impact toughness reaches 21.2 J/cm^2^, being 6.2 times that of the common composite and the highest strength being 67% higher. The pressure infiltration method possesses adaptability to varying shapes and sizes of the products, allowing for large-scale preparation. Therefore, for the first time, the combination of pressure infiltration preparation and architectural design was applied to TiCp/steel composites.

## 1. Introduction

Particle-reinforced metal matrix composites (PRMMCs) [1] have been widely used in aerospace, defense, transportation, mining, power, and other fields owing to their high modulus, high strength, low thermal expansion, and excellent manufacturing flexibility [2,3]. To meet increasing engineering demands, there is an urgent need for high-performance structural materials with strength–toughness matching [4]. However, it is generally accepted that there is a trade-off between the strength and toughness of metal materials [5]. One widely-applied approach to overcome the conflict between strength and toughness is the specific structural design of metallic grains, including laminated structures [6], gradient structures [7], harmonic structures (i.e., bi-modal structures) [8], and their combinations, etc. Another method is through composite architecture (i.e., spatial non-uniform distribution of reinforced particles) design. Joshi et al. [9] proposed a framework where a level-I composite is integrated with an additional phase to form a level-II composite (which can be integrated further into a level-III composite, and so on). Hierarchical composites [10,11] are composed of two or more constituent phases, at least one of which is a composite material. The fracture toughness of the composites was improved using a graded composite architecture, with less strength loss. Zhang et al. [12] prepared bio-inspired graphene/Al_2_O_3_ double-reinforced aluminum composites. Compared to pure aluminum, the hardness, ultimate strength, Young’s modulus, and toughness of the composites increased by 210%, 223%, 78%, and 30%, respectively. Cui et al. [13] prepared TiB_2_/TiAl matrix composites with a layered reinforcement distribution using an annealing method. The tensile strength reached 402 MPa, and the elongation reached 5.72% at 750 °C, providing a good match between strength and plasticity. Ye et al. [14] prepared B_4_C/5083Al composites with a hierarchical structure; the strength remained almost unchanged, whereas the elongation increased from 0.8% to 2.5%. However, this hierarchical composite architecture is rarely applied in steel matrix composites [15].

Titanium carbide particle (TiCp)-reinforced steel matrix composites (TiCp/steel) combine the excellent ductility of steel and the high strength and hardness of TiC particles, achieving high hardness, strength, and excellent wear resistance that is no less than that of WC-Co cemented carbides [16]. TiCp/steel composites are conventionally produced via powder metallurgy (PM) because a wide range of reinforcement volume fractions and sizes can be used [17], and the solid–state process produces a good cermet interface by avoiding severe interfacial reactions [18,19]. However, the preparation of steel matrix composites by PM has the disadvantages of poor adaptability to the shape and size of parts and the need for welding with other parts for use [20]. Pressure infiltration [21,22] has recently emerged as a technique for preparing TiCp/steel composites. OH et al. [23] prepared TiCp/steel composites using pressure infiltration technology. The strength of the composites formed by hot isostatic pressing (919 MPa) was 16% higher than that of the composites formed by infiltration (791 MPa). Therefore, pressure infiltration can achieve high ceramic fraction and superior performance TiCp/steel composites, and the preparation efficiency is higher than that of the PM method. In this paper, the liquid cast–infiltration method is further developed to prepare a new spherical hierarchical architectured composite, and a new preparation method of spherical hierarchical composite preform is proposed.

In the structure of the hierarchical composites, ceramic particles are first uniformly dispersed in the metal matrix to form level I composites, which are then uniformly dispersed in the metal matrix to form level II composites [9]. Generally, the hierarchical composites are fabricated by the PM method. This study adopted an innovative pressure infiltration process. In the process, TiCp preform microspheres with a diameter of 60 μm were first prepared by a spray-drying technique, then the microspheres were put into PVA water solution to make a slurry, and the preforms were prepared by pouring the slurry into a mold. Finally, the composites were prepared by pressure infiltration. The process overcomes the shortcomings of PM methods, realizes the hierarchical distribution of TiCp in the matrix, and improves the comprehensive performance of the strength and toughness of the composite.

## 2. Experimental Procedures

### 2.1. Alloy Preparation

**Figure 1:**
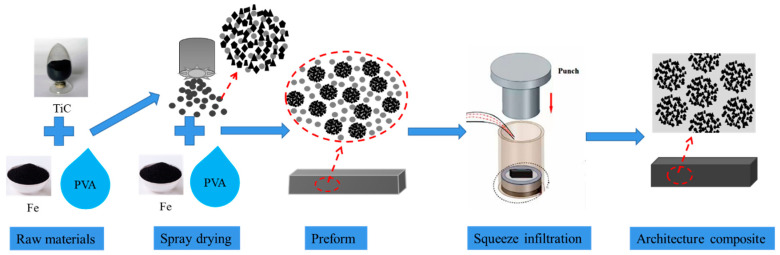
Schematic of preparation of composite.

**Figure 2:**
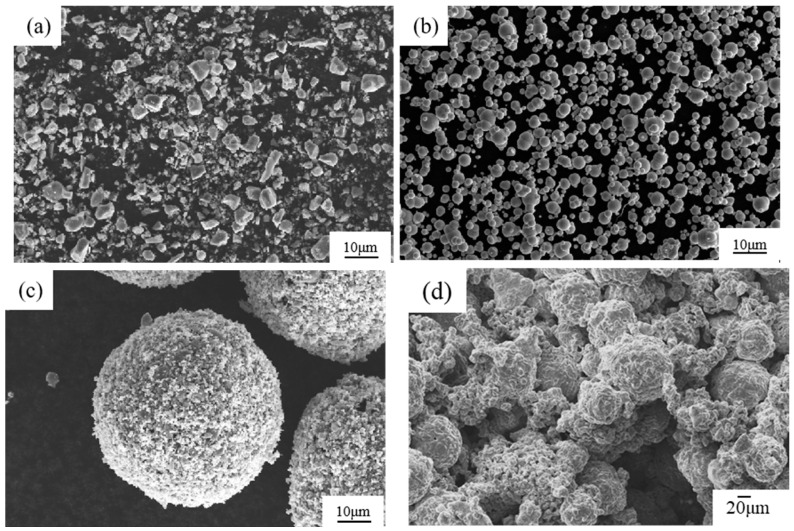
SEM micrographs of TiC particles, Fe particles, ceramic composite microspheres, and TiCp preform. (**a**) Raw TiC particles; (**b**) raw Fe particles. (**c**) Ceramic composite microspheres prepared via spray drying; (**d**) TiCp preform.

Figure 1 presents a schematic diagram of the process for preparing the composites. The reinforcement material is TiC particles (TiCp) with a purity of 99.9% (Qinghe Yuanyao Alloy Products Co., Ltd., Xingtai, China, 1250 mesh), with an average size of 10 μm. The morphology is shown in Figure 2a. The reduced iron powder was used to adjust the mass fraction of TiC in the composites (as shown in Figure 2b). Polyvinyl Alcohol (PVA) aqueous solution with a mass fraction of 40% was selected as the binder. Finally, manganese steel was selected as the matrix, whose chemical composition was C 1.4%, Mn 10%, Cr 2.0%, Si 0.4%, S < 0.05%, *p* < 0.1%, with Fe balance.

The preparation process of the composites is described as follows. First, TiCp, iron powder, and an alcohol solvent were mixed in a mass ratio of 6:4:1 and ball-milled in a planetary ball mill for 3 h (ball-to-material ratio of 5:1, ball diameter of 10 mm, rotation speed of 30 r/min), sprayed by a centrifugal spray-drying method to obtain ceramic microspheres with an average diameter of 60 μm, as shown in Figure 2c. Second, the ceramic microspheres were mixed with iron powder, a binder (40% PVA), and pure water to obtain a slurry. The slurry was poured into a sample mold (70 mm × 50 mm × 20 mm) to form a TiCp preform. The preform was dried and roasted in a vacuum furnace, with the temperature increasing at a rate of 4 °C/min, up to 900 °C, and held for 30 min. After roasting, the preform had enough strength to endure the infiltrating pressure of the steel liquid, due to the sintering of the iron powders in the preform, as shown in Figure 2d.

Manganese steel was smelted in a medium-frequency induction furnace. The TiCp preform was fixed in the casting mold in advance. The molten steel at a temperature of 1550 ± 10 °C was poured into the mold, pressed, and infiltrated into the preform under a pressure of 50 MPa for 5 min. After cooling and solidification, the architectured composite was obtained.

After fabrication, the hierarchical composites were subjected to water-toughening heat treatment, in which the austenitizing temperature was 1050 °C, the holding time was 120 min, and the quenching water temperature was 20 °C. 

In the preparation of the preform, three ceramic microspheres with different mass fractions of 30%, 40%, and 50% were added to obtain the hierarchical composites. For comparison, manganese steel matrix composites reinforced with uniformly distributed TiCp (called common composites) with similar mass fractions and matrix alloy manganese steel were prepared using the pressure casting method with the same parameters.

### 2.2. Structural and Chemical Composition Characterization

The microstructures of the TiC powders, preforms, and composites were observed by scanning electron microscopy (SEM, LEO-1450, SEMTech Solutions, North Billerica, MA, USA). The volume fractions of TiCp and grad II reinforcement in the composite were calculated from five SEM images using Image J software (FIJI), and the average volume fraction was obtained. The phases of the matrix alloy and composites were characterized using X-ray diffraction (XRD; D MAX-2500, Rigaku, Tokyo, Japan), with Cu-Kα (λ = 0.154 nm) radiation in the 2θ range of 1–30° at a scanning rate of 90°/min. The morphology and chemical composition of the TiC/Fe interface were characterized using transmission electron microscopy (TEM, JEM200, JEOL, Frenchs Forest, NSW, Australia). The TEM samples were prepared using a focused ion beam (FIB, ZEISS Crossbeam 540, Macquarie Park, NSW, Australia) system. The elemental distributions of the matrix alloy and composites were studied using electron probe microanalysis (EPMA; JXA-iHP200F, JEOL, Frenchs Forest, NSW, Australia).

### 2.3. Property Tests

The samples for mechanical property tests were cut from composite samples by wire cutting and polished. The dimensions of the samples for the bending strength tests were 5 mm × 5 mm × 30 mm. The bending strength tests were performed using a CMT4503 electronic universal testing machine (Shenzhen Xinsansi material testing Co., Ltd., Shenzhen, China). The test span was 20 mm, and the loading speed was 0.5 mm/min. The impact toughness specimen was sized at 10 mm × 10 mm × 50 mm with no notch. Impact toughness tests were performed using a PH750 impact testing machine (Walter + Bai AG, Nuremburg, Germany). An HR-150A Rockwell hardness tester was used for the hardness test. The data for bending strength and impact toughness were the average of three tests, and the hardness was the average of five tests.

## 3. Experimental Results

(1) Phases of the composites

**Figure 3:**
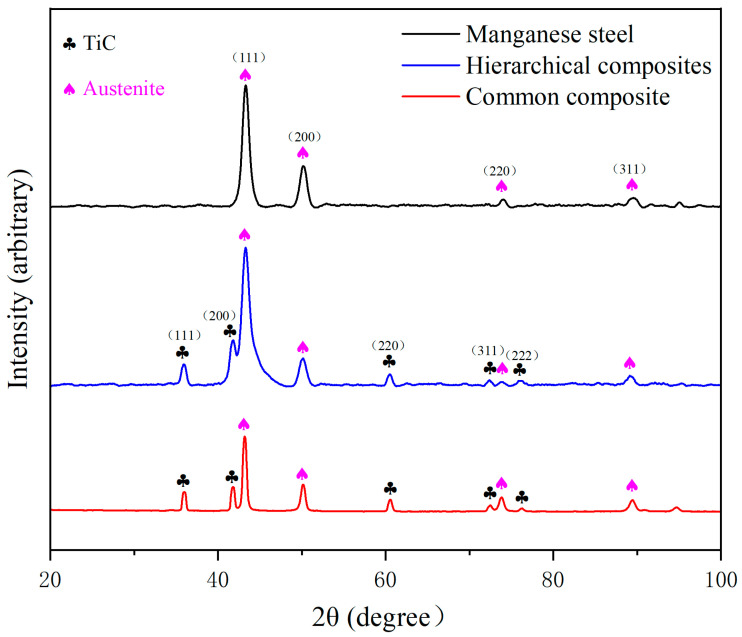
XRD patterns of manganese steel and TiCp/Fe hierarchical composites.

The XRD patterns of the manganese steel and TiCp/Fe common composite and hierarchical composite prepared via pressure infiltration are shown in Figure 3. The microstructure of the manganese steel is comprised of a single austenite phase, and the composites contain both austenite and TiC phases, without other obvious phases.

(2) Microstructure of the composites

**Figure 4:**
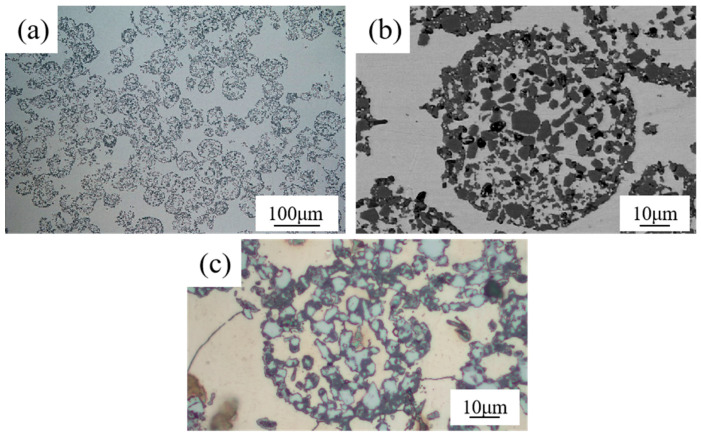
Microstructure of 40% TiCp/Fe hierarchical composites by the pressure infiltration method: (**a**) at low magnification, (**b**) at high magnification, (**c**) matrix inside and outside a composite ball (etched).

Figure 4a shows the microstructure of the TiCp/Fe hierarchical composites prepared by pressure infiltration. The composite is reinforced with uniformly distributed composite balls (level II reinforcement), which is further reinforced with uniformly distributed TiCps, acting as level I reinforcement. So, the composite is a two-level hierarchical composite. Regardless of the level I reinforcement or level II reinforcement, there is no obvious agglomeration. According to quantitative metallographic calculations with Image J, the calculated volume fractions of the three TiCp phases in the hierarchical composite, with mass fractions of 30%, 40%, and 50%, are about 34.8%, 46.3%, and 56.4%. respectively. This hierarchical structure was expected to ensure that the strain was evenly distributed in the composite and prevent strain localization [24,25]. Figure 4b shows a composite ball in the hierarchical composite, revealing that the steel liquid completely infiltrated the TiCp microspheres. Kaplan et al. [26] fabricated TiC-1080 steel cermets by pressure-less infiltration. Small defects were often found at the metal–ceramic interface, and quasi-static failure occurred owing to the extension and passivation of these interface defects. However, in this study, no interfacial defects are observed at the TiC–matrix interface because of the high infiltration pressure.

**Figure 5:**
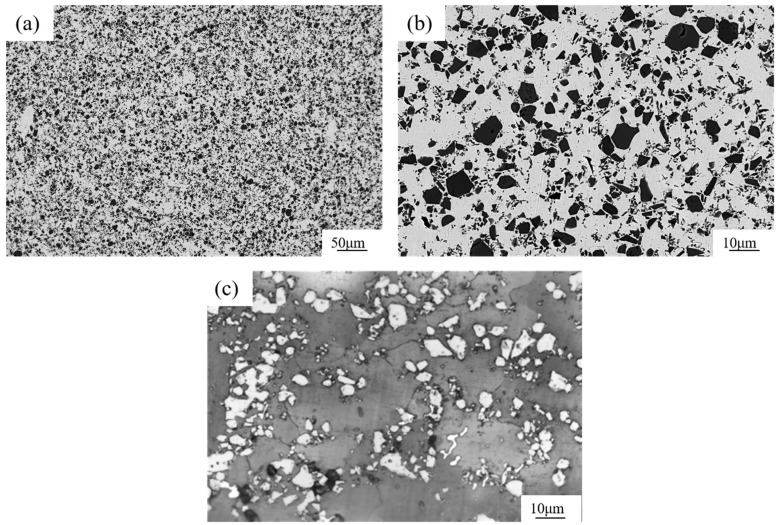
Microstructure of 40% TiCp/Fe common composite prepared by pressure infiltration method: (**a**) at low magnification; (**b**) at high magnification, (**c**) the matrix (etched).

For comparison, the microstructure of the TiCp-reinforced manganese steel matrix composite with a uniform distribution of 46.3% TiCp is shown in Figure 5. There was also no agglomeration of TiCp in the matrix. The TiCp/Fe interface was sound without defects such as pores, cracks, or spalling.

**Figure 6:**
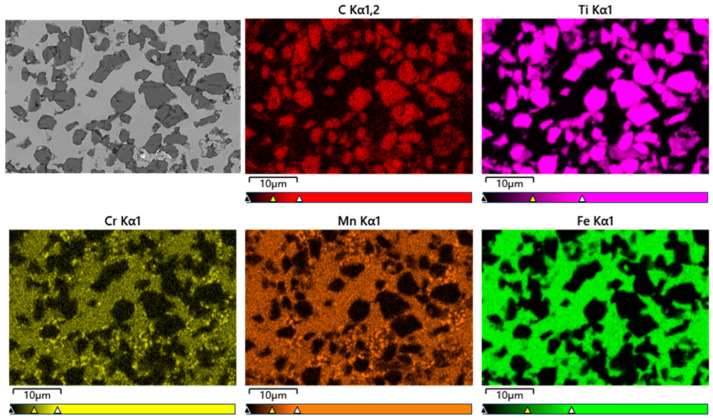
SEM images and elemental distribution of ceramic composite microspheres in TiCp/Fe hierarchical composites.

The microstructure inside the composite balls was further characterized. Figure 6 shows the microstructure and elemental distribution of the composite microspheres in the TiCp/Fe hierarchical composites. The dark-colored particles are TiCps, and the light-colored matrix is manganese steel. Cr and Mn were distributed in the matrix.

**Figure 7:**
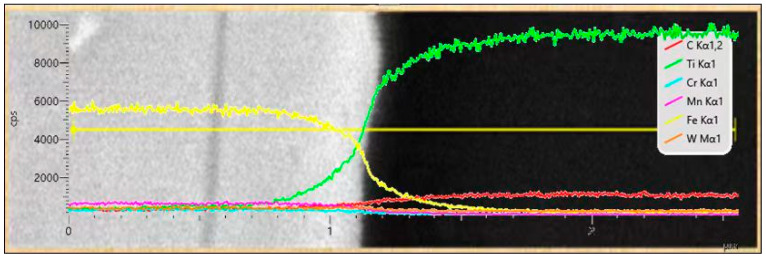
Composition distribution of composite material.

The TiCp/Fe interface was carefully characterized with EDS and TEM. Figure 7 shows the elemental distribution at the interface between the TiCp and the matrix. Ti and C in the TiCp and Fe in the matrix gradually diffused at the interface transition layer to form a good interface bond. The interface transition layer was 2~5 μm thick.

**Figure 8:**
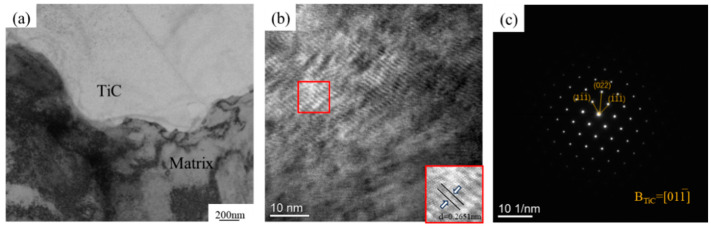
Interfacial microstructures of the hierarchical composite: (**a**) TEM bright–field image of the composite; (**b**) high-resolution (HR) TEM images of TiC–steel matrix interface; (**c**) SADP of TiC particles.

TEM was used to observe the interfacial microstructures of the hierarchical composites, as shown in Figure 8. Figure 8a shows the morphology of the TiCp/Fe matrix interface, indicating that no other phase is found at the interface. Figure 8b shows a high-resolution (HR) TEM image of the TiC–steel matrix interface, demonstrating that the interface between the TiC particles and the matrix was well-bonded. These interface characteristics are the same as those of pressure–infiltration composites in the literature [27]. Figure 8c shows the selected electron diffraction (SADP) pattern of TiCp at the interface, indicating that TiC has a face-centered cubic (FCC) structure.

(3) Chemical composition of the matrices

The composition of the matrices inside and outside the composite balls is different from that of steel liquid before infiltration, because it was changed by the iron powder, which was added to the TiCp preform to adjust the volume fraction of TiCp during the preparation of the hierarchical composite. This means that the chemical composition of the matrix is decided by that of the molten steel and the content of the iron powder in the preform. For example, the mass percentage of the Mn and C element in the matrix inside the composite ball (level I composite) can be calculated by a formula, as follows:

(1) $$\begin{array}{c} wt\%\;\left(Mn\right)=\frac{w\left(Mn\right)}{w\left(C\right)+w\left(Fe\right)+w\left(Mn\right)+w\left(Cr\right)+w\left(Ti\right)}\times 100\% \end{array}$$

(2) $$\begin{array}{c} wt\%\;\left(C\right)=\frac{w\left(C\right)}{w\left(C\right)+w\left(Fe\right)+w\left(Mn\right)+w\left(Cr\right)+w\left(Ti\right)}\times 100\% \end{array}$$

where w(Mn), w(C), w(Fe), w(Cr), and w(Ti) mean the mass of Mn, C, Fe, Cr, and Ti in the matrix, respectively.

**Figure 9:**
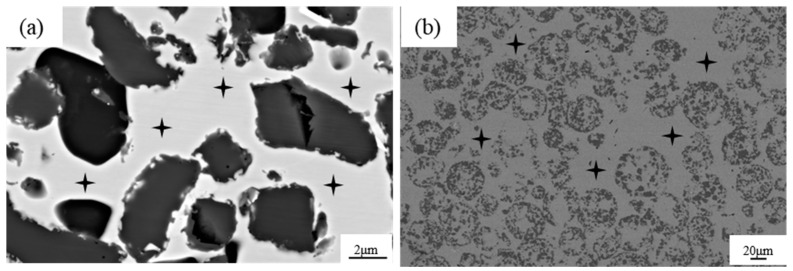
The 40% TiCp/Fe hierarchical composite elemental distribution in the level I matrix and level II matrix: (**a**) level I matrix selection area; (**b**) level II matrix selection area.

The real distribution of elements in the level I and level II matrices was characterized using an electron probe microanalyzer (EPMA). The results are shown in Figure 9 and Table 1, where each value is the average of the five points checked. Figure 9a,b shows the morphologies and dot positions of the level I matrix and level II matrix, respectively. From Figure 9 and Table 1, the Mn content in the level I matrix and level II matrix was approximately 7%, and the C content was 0.569% in the level I matrix and 0.90% in the level II matrix, respectively. Compared with manganese steel before infiltration, Mn and C obviously decreased. This verifies that the manganese steel melt was diluted during infiltration into the TiCp preform containing iron powder, and the dilution degrees are different between the level I matrix and the level II matrix due to different iron powder contents. 

The above-mentioned results show that the content of alloying elements in the matrix of the hierarchical composite will vary according to the metal powder added to the preform through the pressure infiltration method. This requires calculating and adjusting the chemical composition of the matrix in the hierarchical composite from the infiltration alloy in the design of composite materials. 

Furthermore, it was found that the content of Ti in the level I matrix reaches 0.962%, although there is no Ti in the manganese steel melt for infiltration. This indicates that partial dissolution occurred at the surface of TiCp in the steel melt during the infiltration process.

(4) Mechanical properties of the composites

The mechanical properties of the TiCp/Fe composites and the manganese steel are listed in Table 2. The hardness of the hierarchical composites increases with the mass fraction of TiCp from 30% to 50%, and the maximum hardness is 60.7 HRC. However, with the increase in TiCp, the impact toughness and bending strength of the hierarchical composites show an increasing trend first and then decrease; the highest values are obtained at 40% TiCp.

Compared with the manganese steel and the common TiCp/Fe composite, the highest hardness (60.7 HRC) of the hierarchical composites is about 3.1 times that of the manganese steel (19.3 HRC), and similar to that of the common TiCp/Fe composite (56.9 HRC), with the same TiCp content. Compared with the literature [28], where the hardness of the 36% TiCp-reinforced steel composites prepared by PM was approximately 70 HRC, the hardness of the same composite prepared by the pressure infiltration method is slightly lower. 

With TiCp content increasing, both the bending strength and the impact toughness first increase, then decrease, and reach the highest values at 40% TiCp. The highest strength of the TiCp/Fe hierarchical composite is 908.0 MPa, increasing by 48.9%, compared with the strength (609.8 MPa) of the common composite. The highest impact toughness of the hierarchical composites is 21.2 J/cm^2^, reaching about 6.2 times that of the common composite (3.4 J/cm^2^). That is to say, both the bending strength and impact toughness of the TiCp/Fe hierarchical composites are significantly higher than those of the common composite. Therefore, the hierarchical spatial design significantly improves the comprehensive properties of strength and toughness of the TiCp/Fe composites. Although the bending strength and impact toughness of the hierarchical composites are both lower than that of the manganese steel matrix, the composites often exhibit superior wear performance as wear-resistant materials [29]. The wear resistance of the hierarchical composites will be researched next in this work.

**Figure 10:**
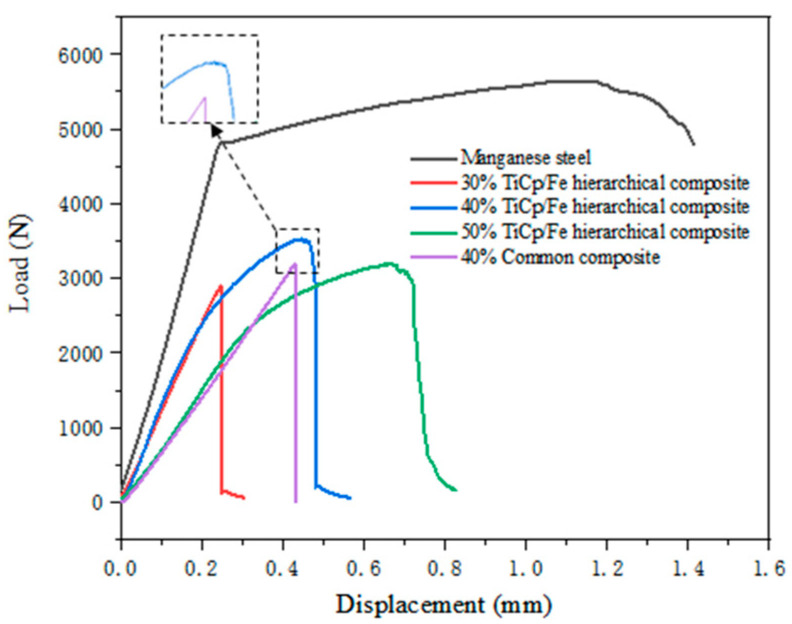
Bending load–displacement curves of TiCp/Fe composites and manganese steel matrix.

Figure 10 shows the bending load–displacement curves of the TiCp/Fe composites and the manganese steel matrix. It can be observed that the plastic deformation stage in the curves of the hierarchical composites is very obviously present, especially for those containing 40% and 50% TiCp; however, there is no plastic deformation stage for the common composite. The curve comparison proves that the hierarchical composites compose much better plasticity than the common composite. In the locally magnified image of the curve of the 40% TiCp hierarchical composite, it can be observed that the curve fluctuates in a zigzag manner around the highest load, which does not occur in the common composite. The difference will be further analyzed in the discussion section.

## 4. Discussion

(1) Influence of hierarchical structure on the mechanical properties of the TiCp/Fe composites

The multicore architecture, also known as a concrete-like architecture, offers both significant toughness and strengthening effects simultaneously due to the reduction of the interface volume density between the soft and hard phases [30]. However, in the multicore architectured composite, the hard phase regions are shaped into fibers, so there is stress concentration at the ends, which can easily lead to crack formation. Therefore, if the shape of the hard phase regions is changed to spheres, the stress concentration phenomenon will be completely eliminated. For example, Kou et al. [31] studied a composite with a spatial lattice architecture and spherical hard phase regions at the millimeter scale. The results show that the strain of the hierarchical composites reaches six times higher than that of the common composite, with a yield strength 79.5% higher. However, the hard phase regions are too coarse, so it is not beneficial to eliminate strain localization at the interface between the hard phase region and the soft phase region, not improving the comprehensive performance of the composites. 

In this work, spherical hierarchical composites were prepared with much smaller-sized hard phase regions of about 60 microns, compared to those in the literature. As a result, the bending strength of the 40% TiCp/Fe hierarchical composite becomes 67% higher than the common composite. Especially noteworthy is its impact toughness, which reaches 21.2 J/cm^2^, being 6.2 times that of the latter. The strengthening and toughening effect of the spherical hierarchical architecture is obvious.

**Figure 11:**
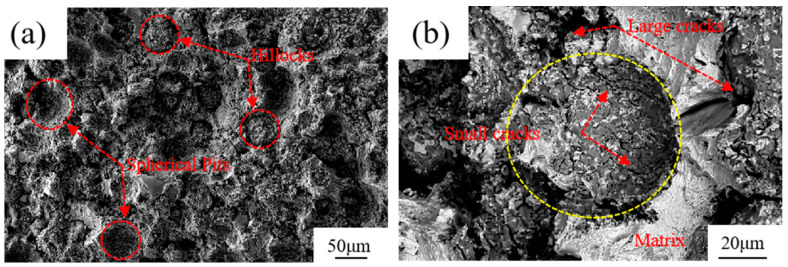
The 40% TiCp/Fe hierarchical composite bending specimen fracture: (**a**) macroscopic morphology of fracture; (**b**) micro-morphology of fracture.

The fracture behavior of the TiCp/Fe hierarchical composites can be explained by the fracture morphology of the composites under the bending load, as shown in Figure 11. Figure 11a shows many spherical pits and hillocks on the fracture surface, and Figure 11b shows that large cracks propagate and are deflected along the level II reinforcement/level II matrix interface. Although there are also many small cracks in the composite ball like those in Figure 11b, they seldom evolve into main cracks and cause the composite balls to crack. The situation is the same for the fracture surface analysis of the impact samples. Therefore, under the case of static bending or high-speed impact load, cracking of the level II reinforced ball/level II matrix are the main failure mechanisms of the hierarchical composites [32]. Owing to the continuous deflection of cracks along the curved surfaces of different composite spheres, more energy is released during crack propagation, which explains the high toughness of the TiCp/Fe hierarchical composites.

**Figure 12:**
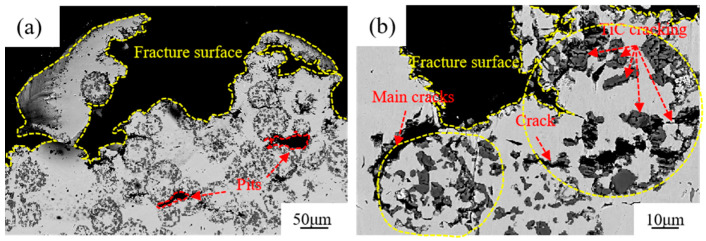
SEM micrographs of the subsurface layer of the 40% TiCp/Fe hierarchical composite cross-section after the bending strength test: (**a**) macroscopic morphology of surface; (**b**) micro-morphology of surface.

To carefully analyze the origin and propagation of cracks in the hierarchical composites, the fractured samples were coldly inlaid in resin and cut longitudinally, and the morphology under the fracture surfaces was observed, as shown in Figure 12. As shown in Figure 12a, the fracture surface of the composite extends along the contour of several level II reinforcements, and two obvious cracks originate at the connection regions of adjacent level II reinforcements. Figure 12b shows the morphology of the subsurface layer of the fractured surface at a high magnification. Because TiCp is brittle, many microcracks first form inside TiCps, linking by breaking the level I matrix between them at both the interface of level II reinforcement/matrix and that of the level I reinforcement/matrix. The cracks at the interface of level II reinforcement/matrix can link together and form a main crack to propagate to the fracture surface; however, the crack inside the level I composite cannot. The reason should lie in the difference of stress distribution between inside and outside the level II reinforcement. According to the literature [33], the maximum stress of hierarchical composites is located at the interface of level II reinforcement/matrix, rather than at the ceramic particle/matrix interface inside level II reinforcements. 

As shown in Figure 10 above, from the locally magnified image of the curve of the 40% TiCp hierarchical composite, it can be observed that the curve fluctuates in a zigzag manner around the highest load, which does not occur in the common composite. Combined with the fracture surface of the 40% TiCp/Fe hierarchical composite material, it is found that the material at the end of the fracture failure is due to the spatial structure of the hierarchical configuration, leading to the zigzag fluctuation on the bending load–displacement curves.

**Figure 13:**
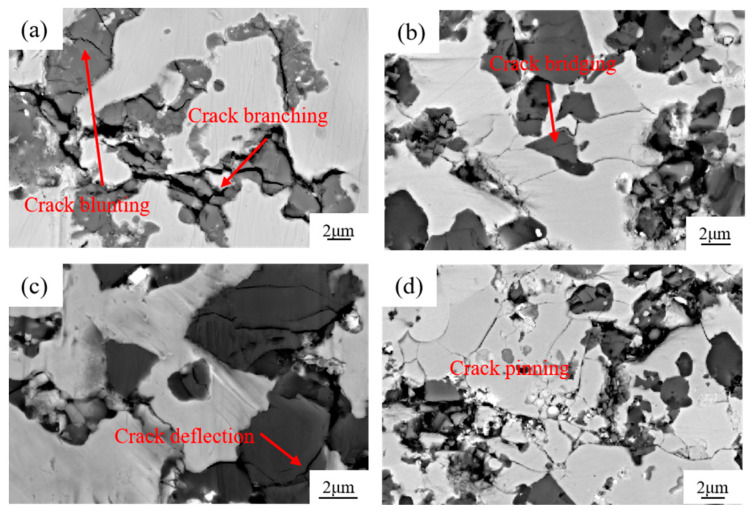
SEM micrographs of cracks in reinforcement particles: (**a**) crack branching; (**b**) crack bridging; (**c**) crack deflection; (**d**) crack pinning.

The cracking behavior of TiCp and the adjacent matrix observed in this research mainly includes crack deflection [34], crack bridging [35], crack bifurcation [36], and the pinning phenomenon [37,38], as shown in Figure 13. In Figure 13a, cracks inside TiCp due to its brittleness are hindered at the TiCp/Fe interface. In Figure 13b, TiC particles connect two tiny branch cracks with different directions to form a bridging effect. The crack deflection phenomenon is widely present at the interface of level II reinforcement/matrix and level I reinforcement/matrix, as seen in Figure 13c. When interfaces are formed with TiCps and other phases, small dislocations around them will cause a pinning effect [36]. This pinning effect makes it difficult for the early-generated micro-opening to move and prevents its convergence and connectivity with the main opening, requiring more energy to achieve convergence and connectivity (as shown in Figure 13d). These cracking behaviors also contribute to the high toughness of the TiCp/Fe hierarchical composites.

(2) Innovative method to prepare the hierarchical composites—pressure infiltration

Generally, the method of preparing architectured composites is still powder metallurgy (PM) [14], the same method as conventional composites. However, the preparation process requires multiple mixing and multiple sintering, so there are disadvantages such as a long process, low efficiency, and limitations on the shape and size of the products. Therefore, various liquid infiltration methods have become a hot spot in the preparation of architectured composites in recent years. Qin et al. [30] drilled holes in an Al block, then filled the mixed powders of ceramic and matrix metal into the holes, and finally prepared the multi-core architectured composite using vacuum pressure infiltration technology. Obviously, this method is only suitable for research because the drilling process greatly reduces the preparation efficiency. Lu et al. [39] prepared templates of plastics containing the needed architecture by 3D printing, then filled them with mixed powders of ceramic and base metal, and finally prepared three-dimensional interpenetrating network composites by pressure infiltration technology. This method can improve the accuracy of the architecture but can only be applied to architectured composites where the soft phase regions and the hard phase regions are interpenetrating. It cannot be used for architectured composites where one of the soft phase regions and the hard phase regions are continuous and the other is dispersed. For this purpose, Kou et al. [31] connected several ceramic preform balls in series with a steel wire to form a sugar-gourd skewer, which was placed in a casting cavity to finally prepare a spatial lattice architectured composite by pressure infiltration technology. Obviously, this method can only be applied in research and does not have feasibility for large-scale preparation. Additionally, the hard phase regions must reach the size of millimeter level, which is not beneficial to the structural refinement of the composites.

The present paper proposes an innovative process for the large-scale preparation of composites with a spherical hierarchical architecture, in which soft phase regions are continuous and spherical hard phase regions dispersed. First, tiny balls containing mixed particles of ceramic and metal, with an approximate diameter of 60 microns, were formed by spray drying technology. Then, the balls were mixed with metal powder to obtain preforms with the desired shape and size. Finally, the pressure infiltration method was employed to fabricate the architectured composites in one step. This method effectively solves the challenges encountered in powder metallurgy methods such as poor adaptability to varying shapes and sizes of composites, as well as difficulties associated with large-scale preparation observed in various methods only for research. Moreover, it enables refinement of the size of the hard phase regions down to a 10-micron scale, thereby facilitating further enhancement of the mechanical properties of the composites.

In summary of this study, the spherical hierarchical architecture design was first applied to the TiCp/steel composites. A new preparation process for hierarchical composites was proposed so that mass production and wide applications of TiCp/steel hierarchical composites became feasible. However, there are still some problems, such as the surface oxidation of TiCp during the preparation and the mechanical properties of the matrix itself (the strength and hardness of the manganese steel matrix are too low to support TiCp during application), which need to be further solved in future research.

## 5. Conclusions

Spherical TiCp/Fe hierarchical composites were successfully prepared via pressure infiltration technology. The strengthening and toughening mechanisms of the composites were studied by extensively characterizing their microstructures and mechanical properties. The main conclusions of this study are summarized as follows: 

(1) A new pressure infiltration technology was successfully developed to prepare spherical TiCp/Fe hierarchical composites. 

(2) With the increase inTiCp fraction, the hardness of TiCp/Fe hierarchical composites gradually increases, and the impact toughness and bending strength increase first and then decrease. The highest impact toughness reaches 21.2 J/cm^2^, being 6.2 times that of the common composite, with the highest strength being 67% higher.

(3) High-resolution (HR) TEM images of the TiCp/steel matrix interface revealed that the partial dissolution and reprecipitation of TiC at the TiCp/Fe interface during pressure infiltration improve wettability, leading to interface stability and strong adhesion between the reinforced material and the matrix.

(4) The high toughness of the TiCp/Fe hierarchical composites can be attributed to various fracture mechanisms, including crack passivation, crack branching, crack deflection, matrix plastic deformation, and crack bridging.

## Figures and Tables

**Table 1:** The 40% TiCp/Fe hierarchical composite element content in the matrix.

Element	Manganese Steel (wt%)	Level I Matrix (wt%)	Level II Matrix (wt%)
C	1.3	1.0	1.0
Ti	0.0	1.0	0.0
Cr	2.0	2.0	1.7
Mn	8.7	7.0	7.1
Fe	88.0	88.3	89.0

**Table 2:** Mechanical properties of researched materials.

Materials	Hardness (HRC)	Impact Toughness (J/cm^2^)	Bending Strength (MPa)
Manganese steel	19.3	290.0	1420.2
Common composite (40% TiCp)	56.9	3.4	609.8
Hierarchical composite (30% TiCp)	52.9	15.0	711.6
Hierarchical composite (40% TiCp)	57.0	21.2	908.0
Hierarchical composite (50% TiCp)	60.7	19.9	765.5

## Data Availability

The data that have been used are confidential.
